# The silent assassin: Third hand smoking

**DOI:** 10.7189/jogh.12.03079

**Published:** 2022-12-03

**Authors:** Delfin Lovelina Francis, Saravanan Sampoornam Pape Reddy

**Affiliations:** 1 Department of Public Health Dentistry, Saveetha Dental College & Hospitals, Saveetha Institute of Medical and Technical Sciences (SIMATS), Saveetha University, Chennai, Tamilnadu, India; 2 Department of Periodontology, Army Dental Centre (Research & Referral), Army Hospital, New Delhi, India

Never-smokers are being diagnosed with lung cancer at an alarmingly increasing rate in Asian countries [1]. This trend is a major cause for concern. Lung cancer has been attributed to genetic susceptibility, occupational hazards, poor air quality due to pollution, second-hand smoking, however the logical explanation for third hand smoking (THS) is not well established. It has been suggested that second-hand smoking is the most important factor [2]. Interestingly, a single centre study found that, there is no difference in the likelihood of lung cancer survival between people who have smoked throughout their lives and those who have never smoked [3]. A recent article published in the Lancet examined the patterns and burden of chronic lung diseases over the past nearly twenty years and listed both smoking and passive smoking as risk factors. However, there is data that contradicts this, suggesting that there is a lesser likelihood of getting lung cancer from second-hand smoke (SHS). Because of this, the question of whether or not oxidative DNA damage can be caused by SHS has been brought into question [4].

Third hand smoking is defined as the persistent residue produced by aged second-hand smoke that adheres to indoor dust and surfaces and reemits into the air, posing a public health risk. The characteristics of THS are being universally present, having a nature that has persisted for a long time and being unconscious to the population that is being affected by it. It is a way of thinking about exposure to tobacco and its products that is indirect and takes place over a longer period of time. However, the most significant distinction lies in the fact that THS includes not only the inhalational route but also the ingestive route and topical route. THS can affect people of all ages, from infants to the elderly, and it is a major risk factor and contributor to the development of a variety of cardiovascular and pulmonary conditions, including lung cancer [5]. Another intriguing discovery was that people who had never smoked had a higher incidence of lung cancer than people who smoked, suggesting that there is a third factor in addition to active and passive smoking [6].

## PATHODYNAMIC MECHANISMS OF THIRD HAND SMOKING

THS and its relationship to lung cancer warrants greater scientific inquiry, as very little is known about it at this time. This is in contrast to the well-established link between SHS and lung cancer. A recent study based on self-reporting questionnaires found that, subjects who were exposed to third-hand smoke had significant levels of salivary cotinine similar to the individuals exposed to SHS [7]. Therefore, third-hand smoke is considered a potential ubiquitous predisposing factor for lung cancer and chronic lung diseases. This is highly unrecognized among medical professionals and is presenting a grave ongoing public health hazard given that third-hand smoke is considered to be omnipresent. This in and of itself raises a significant issue regarding the validity of non-smoker status in the well-established literature that has been published in the past. The segment of the population that is totally shielded from exposure to third-hand smoke is going to be an area of focus in forthcoming research related to tobacco smoking and related topics [8].

**Figure:**
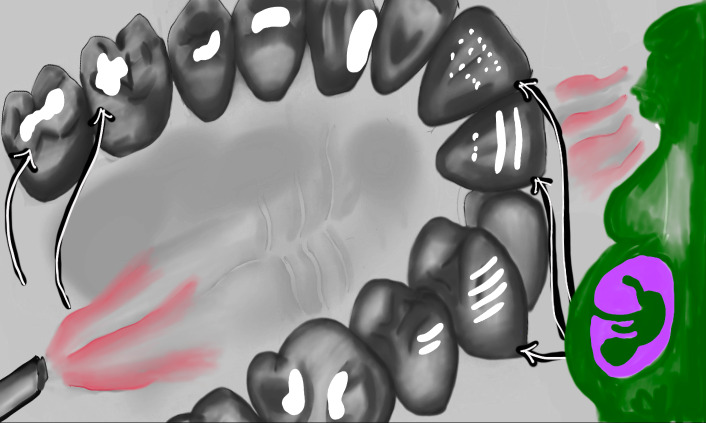
Photo: Potential effects of third hand smoking. Source: Self prepared by authors.

Traditional smoking results in increased exposure to SHS and THS because of the formation of harmful pyridinyl radicals (high potential species related to lung cancer) as a result of thermal dissociation kinetics of nicotine at higher temperatures. However, smoking devices of the third generation, which operate at temperatures that are significantly lower than those of their predecessors, are said to be less hazardous. Tobacco use has been extensively researched and validated as a possible contributor to tobacco use syndrome and THS, whereas the same cannot be said for electronic cigarettes [9]. Additionally, it was demonstrated that the use of heat-not-burn tobacco products led to a significant decrease in the function of the vascular endothelial cells and induced an excessive amount of oxidative stress. Even though there was a significantly lower amount of nitrosamine emissions in comparison to traditional tobacco smoking, it was discovered that they were still detrimental to the cardiovascular health [10].

The biomonitoring of tobacco exposure research is the target biomarker for tobacco-specific biomarkers as well as biomarkers that are related to tobacco use [11]. Over the course of several decades, nicotine and its by-products were analysed from urine, serum, saliva, and gingival crevicular fluid in order to characterize the type of exposure, its extent, and its nature. However, none of these have been validated and demonstrated to be an accurate tobacco biomarker. On the other hand, the confirmation of the same for subjects who were exposed to third-hand smoke is almost non-existent in the literature that is currently available.

When it comes to third-hand smoke, just as there are no safe levels of exposure to second-hand smoke, the same goes for THS, which is completely hidden and requires research with rigorous criteria. In addition to this, there is a notable trend that points to a decline in the incidence of lung cancer that can be attributed to second-hand smoke as well as a reduction in the number of deaths that are caused by second-hand smoke. On the other hand, there was a significant rise in the number of cases of lung cancer that were caused by factors other than smoking. This was the case on the other end of the spectrum. The majority of studies that are conducted in the field of public health make use of an index called the “Estimated Annual Percentage Change” to describe the epidemiological trends in the burden of diseases. However, the vast majority of these studies have only conducted a combined analysis of active and passive smoking to demonstrate an increased risk of developing lung cancer. In addition, the majority of the studies relied on the data that were self-reported and were based on historical recall. When combined with a genetic predisposition, second-hand smoke plays a significant part in the process of tumour angiogenesis, as seen in some cases of breast cancer [12].

## PUBLIC HEALTH CONCERN

Children were found to be particularly susceptible to the effects of any form of passive smoking in the course of a smoke-free school interventional pilot randomized trial [13]. To this day, THS has not been able to be quantified or categorized and there is lack of data to explain the possible relationships between THS and lung cancer using disease progression models. In the field of public health, definitive trials are needed to evaluate the cost-effectiveness of various levels of prevention and health promotion to reduce the risk associated with THS, which has thus far received less attention. This risk can be reduced by decreasing the amount of second-hand smoke that is inhaled [14]. It is time to raise awareness about the potential negative effects of THS exposure and advocate for smoke-free homes for the sake of future generations. A major public health concern that must be addressed is ensuring smoke-free legislation that will bring out relevant strategies to mitigate THS source production and exposures. The policies and legislative regulations on smoking need to be re-examined on a global scale to investigate the potential causes of THS, which could reduce the number of cases that will occur in the future [15]. Although public smoking bans and regulations are in place in the majority of countries around the world, it is expected that rules regarding smoking in the home will become the new standard in order to curb the growing number of potential dangers caused by THS. This suggests that additional studies focusing specifically on investigating the role of THS in pulmonary conditions and lung cancer need to be carried out to shed light on the topic.

## References

[1] Zhou F Zhou C Lung cancer in never smokers-the East Asian experience. Transl Lung Cancer Res. 2018;7:450-63. 10.21037/tlcr.2018.05.14 30225210 PMC6131183

[2] Rivera GA Wakelee H Lung Cancer in Never Smokers. Adv Exp Med Biol. 2016;893:43-57. 10.1007/978-3-319-24223-1_3 26667338

[3] Subramanian J Velcheti V Gao F Govindan R Presentation and stage-specific outcomes of lifelong never-smokers with non-small cell lung cancer (NSCLC). J Thorac Oncol. 2007;2:827-30. 10.1097/JTO.0b013e318145af79 17805060

[4] Krishnan VG Ebert PJ Ting JC Lim E Wong S-S Teo ASM Whole-genome sequencing of asian lung cancers: second-hand smoke unlikely to be responsible for higher incidence of lung cancer among Asian never-smokers. Cancer Res. 2014;74:6071-81. 10.1158/0008-5472.CAN-13-3195 25189529

[5] Moon SY Kim TW Kim YJ Kim Y Kim SY Kang D Public Facility Utility and Third-Hand Smoking Exposure without First and Second-Hand Smoking According to Urinary Cotinine Level. Int J Environ Res Public Health. 2019;16:855-9. 10.3390/ijerph16050855 30857230 PMC6427616

[6] Das A Krishnamurthy A Ramshankar V Sagar TG Swaminathan R The increasing challenge of never smokers with adenocarcinoma lung: Need to look beyond tobacco exposure. Indian J Cancer. 2017;54:172-7. 10.4103/ijc.IJC_33_17 29199684

[7] Lidón-Móyano C Fu M Perez-Ortuno R Ballbè M Garcia E Martin-Sànchez JC Third-hand exposure at homes: Assessment using salivary cotinine. Environ Res. 2021;196:110393. 10.1016/j.envres.2020.110393 33129855

[8] Matt GE Quintana PJE Hoh E Zakarian JM Dodder NG Record RA Remediating Thirdhand Smoke Pollution in Multiunit Housing: Temporary Reductions and the Challenges of Persistent Reservoirs. Nicotine Tob Res. 2021;23:364-72. 10.1093/ntr/ntaa151 32803265 PMC7822102

[9] Chavarrio Cañas JE Monge-Palacios M Grajales-Gonzalez E Sarathy SM Early Chemistry of Nicotine Degradation in Heat-Not-Burn Smoking Devices and Conventional Cigarettes: Implications for Users and Second- and Third-Hand Smokers. J Phys Chem A. 2021;125:3177-88. 10.1021/acs.jpca.1c01650 33834773 PMC8154610

[10] Fried ND Gardner JD Heat-not-burn tobacco products: an emerging threat to cardiovascular health. Am J Physiol Heart Circ Physiol. 2020;319:H1234-9. 10.1152/ajpheart.00708.2020 33006919 PMC7792702

[11] Torres S Merino C Paton B Correig X Ramírez N Biomarkers of Exposure to Secondhand and Thirdhand Tobacco Smoke: Recent Advances and Future Perspectives. Int J Environ Res Public Health. 2018;15:2693. 10.3390/ijerph15122693 30501044 PMC6313747

[12] Global Burden of Disease Cancer Collaboration Fitzmaurice C Abate D Abbasi N Abbastaber H Abd-Allah F Global, Regional, and National Cancer Incidence, Mortality, Years of Life Lost, Years Lived with Disability, and Disability-Adjusted Life-Years for 29 Cancer Groups, 1990 to 2017: A Systematic Analysis for the Global Burden of Disease Study. JAMA Oncol. 2019;5:1749-68. 10.1001/jamaoncol.2019.2996 31560378 PMC6777271

[13] Siddiqi K Huque R Kanaan M Ahmed F Ferdous T Shah S Children Learning About Secondhand Smoke (CLASS II): A Pilot Cluster Randomized Controlled Trial. Nicotine Tob Res. 2019;21:670-7. 10.1093/ntr/nty090 29771390 PMC6468126

[14] Huque R Siddiqi K Smoke-free homes: The final frontier. Tob Prev Cessat. 2021;7:63. 10.18332/tpc/142772 34722952 PMC8519312

[15] Acuff L Fristoe K Hamblen J Smith M Chen J Third-Hand Smoke: Old Smoke, New Concerns. J Community Health. 2016;41:680-7. 10.1007/s10900-015-0114-1 26512014

